# Metabolic Syndrome among Stable Chronic Obstructive Pulmonary Disease Patients Visiting Outpatient Department of a Tertiary care centre: A Descriptive Cross-sectional Study

**DOI:** 10.31729/jnma.8129

**Published:** 2023-04-30

**Authors:** Santosh Baniya, Tirtha Man Shrestha, Pankaj Pant, Ramesh Prasad Aacharya

**Affiliations:** 1 Department of General Practice and Emergency Medicine, Pokhara Academy of Health Sciences, Ramghat, Pokhara, Nepal; 2 Department of General Practice and Emergency Medicine, Institute of Medicine, Tribhuvan University Teaching Hospital, Maharajgunj, Kathmandu, Nepal; 3 Department of Pulmonology and Critical Care, Tribhuvan University Teaching Hospital, Maharajgunj, Kathmandu, Nepal

**Keywords:** *chronic obstructive pulmonary disease*, *c-reactive protein*, *metabolic syndrome*

## Abstract

**Introduction:**

Metabolic syndrome; a constellation of obesity, hypertension, and disturbances of lipid and carbohydrate metabolism is a common phenomenon in chronic obstructive pulmonary disease. Systemic inflammation plays an important role in both conditions. The aim of this study was to find out the prevalence of metabolic syndrome among stable chronic obstructive pulmonary disease patients visiting the outpatient Department of a tertiary care centre.

**Methods:**

A descriptive cross-sectional study was done in the outpatient Department of Pulmonology and General Practice from 1 August 2019 to 31 December 2020. Ethical approval was obtained from Institutional Review Committee [Registration number: 5/(6-11)E2/076/077]. Convenience sampling method was used. Point estimate and 95% Confidence Interval were calculated.

**Results:**

Among 57 patients with stable chronic obstructive pulmonary disease, the prevalence of metabolic syndrome was 22 (38.59%) (27.48-49.70, 90% Confidence Interval). The prevalence of metabolic syndrome in patients with Global Initiative for Obstructive Lung Disease stages 1, 2, 3, and 4 were 6 (27.27%), 9 (40.90%), 6 (27.27%) and 1 (4.54%) respectively.

**Conclusions:**

The prevalence of metabolic syndrome was similar to the other studies done in similar settings. The screening of metabolic syndrome is necessary and stratification for cardiovascular disease risk is important for timely intervention to prevent and decrease morbidities and mortalities.

## INTRODUCTION

Chronic obstructive pulmonary Disease (COPD), a non-reversible obstructive disease of the airways has been associated with a number of extra-pulmonary manifestations like obesity, metabolic syndrome and osteoporosis.^1^ COPD is characterized by systemic inflammation along with airway inflammation and various etiopathogenetic mechanisms; systemic inflammation, physical inactivity, and adipose tissue inflammation have been proposed to describe these manifestations.^2-4^ Metabolic syndrome (MetS); a constellation of obesity, hypertension, and disturbances of lipid and carbohydrate metabolism is a notable extrapulmonary manifestation which contributes to increased morbidity and mortality.^5^

Despite this established fact, there is a paucity of studies focusing on the status of MetS in COPD patients in Nepal so we conducted this study to identify the status of MetS in stable COPD patients.

The aim of this study was to find out the prevalence of metabolic syndrome in stable chronic obstructive pulmonary disease patients visiting the outpatient Department of a tertiary care centre.

## METHODS

A descriptive cross-sectional study was conducted among stable COPD patients visiting the outpatient clinic of the Department of Pulmonology and the Department of General Practice of Tribhuvan University Teaching Hospital, Kathmandu from 1 August 2019 to 31 December 2020 after receiving ethical approval from the Institutional Review Committee [Registration number: 5/(6-11)E2/076/077]. Written informed consent was taken from the participants. Adult patients (>18 years) diagnosed with COPD, not in exacerbation visiting the outpatient clinic were included in the study. Patients in exacerbation as per GOLD criteria, patients with active infections, patients currently smoking and patients with a known malignancy or chronic inflammatory diseases were excluded from the study. Convenience sampling method was used. The sample size was calculated using the following formula:


$$\begin{array}{ll} \text{n} & ={\text{Z}}^{2}\times \frac{\text{p}\times \text{q}}{{\text{e}}^{\text{2}}}\\ & =1.64^{2}\times \frac{0.357\times 0.643}{0.11^{2}}\\ & =52 \end{array}$$

Where,

n = minimum required sample size
Z = 1.64 at 90% Confidence Interval (CI)
p = prevalence of metabolic syndrome was taken from a previous study, 35.71%^6^
q = 1-p
e = margin of error, 11%

Metabolic syndrome was defined using International Diabetes Federation 2006 criteria.^7^ Patients with central obesity (Male >90 cm and Female >80 cm) and any of two among raised triglycerides (≥150 mg/ dl or 1.7 mmol/l) or previously under treatment for hypertriglyceridemia, reduced HDL-cholesterol (<40 mg/dl or 1.03 mmol/L) in males and (<50 mg/dl or 1.29 mmol/l) in females) or previously under treatment for lipid abnormality, raised blood pressure (≥130 mm Hg systolic and ≥85 mm Hg diastolic blood pressure) or previously under treatment for hypertension and raised fasting blood sugar level (>100 mg/dl or 5.6 mmol/l) or previously diagnosed type 2 diabetes mellitus were diagnosed as MetS.

Data were entered in Microsoft Excel Version 2016 and analysed using IBM SPSS Statistics version 25.0. Point estimate and 90% CI were calculated.

## RESULTS

Among 57 stable COPD patients, the prevalence of metabolic syndrome was found to be 22 (38.59%) (27.48-49.70, 90% CI). Out of which, 6 (27.27%) were from the General Practice outpatient clinic and 16 (72.72%) were from Pulmonology outpatient clinic. The mean age of the patients was 68.96±8.06 years (Range: 45-87 years). A total of 44 (77.19%) patients were between 61-80 years and the male:female ratio of the study population was 9:10 (Table 1).

A total of 9 (42.85%) patients in GOLD stage II had MetS, and 6 (50%) in GOLD stage I had MetS (Table 1).

**Table 1:** COPD patients with MetS based on GOLD staging (n= 22).

Stages	n (%)
GOLD Stage 1	6 (27.27)
GOLD Stage 2	9 (40.90)
GOLD Stage 3	6 (27.27)
GOLD Stage 4	1 (4.54)

The mean CRP of COPD patients with MetS is 5.01±0.58mg/L (Table 2).

**Table 2:** Status of CRP in COPD patients with Mets based on GOLD stages (n= 22).

Stages	CRP (mean±SD) mg/l
GOLD Stage 1	4.21±0.41
GOLD Stage 2	4.25±0.72
GOLD Stage 3	4.75±0.60
GOLD Stage 4	4.97±0.73

The mean waist circumference of patients with MetS in male and female were 96.08±2.71 cm and 92.63±6.78 cm respectively. Hypertension 20 (90.90%) was the most common finding followed by 19 (86.36%) elevated fasting blood sugar (FBS) levels, reduced HDL level 9 (40.90%) and increased triglyceride level 7 (31.80%) among the patients MetS.

**Table 3:** Clinical and laboratory parameters among COPD patients with MetS (n= 22).

Variables	mean±SD
BMI (kg/m^2^)	28.5±1.9
Systolic blood pressure (mm of Hg)	133.8±13.0
Diastolic blood pressure (mm of Hg)	86.2±6.7
Fasting blood glucose (mmol/l)	6.20±0.90
HDL (mmol/l)	1.20±0.30
Triglyceride (mmol/l)	1.50±0.60

## DISCUSSION

In our study, 38.59% of stable COPD patients had coexisting MetS. In a study done in Germany using IDF criteria^7^ for diagnosis of MetS, the prevalence was 47.5%.^8^ Similarly, a study conducted, using IDF criteria found a prevalence of 37.8%.^9^ On the other hand, a similar study using ATP III criteria^10^ and excluding patients with diabetes, cardiovascular disease and other co-morbidities found a prevalence of 21%.^11^ And, in a study in Egypt MetS was present in 40% of the COPD patients.^12^ The prevalence depends on the criteria used to diagnose MetS along with the geography of where the study is conducted.

Our study concluded that metabolic syndrome is less frequent in the severe form of the disease. More than one-third of the patients (36.8%) were in Gold stage II and only (10.5%) were in Gold stage IV. There was a gradual decline in the prevalence of MetS with an increase in the severity of the disease. In a study, the prevalence of MetS in Gold I-IV was 38.5%, 52.8%, 30%, and 33.3% respectively.^13^ Similarly, another study showed a prevalence of 50%, 53%, 37%, and 44% in Gold stage I-IV.^8^ In a Canadian study the overall prevalence of MetS in patients was 47% and the frequency decreased to about 10% at Gold stage III and IV.^14^ Several studies conducted around the world concluded that the MetS is less frequent in the severe form of the disease and is a consequence of weight loss which occurs in patients with advanced disease.

C-reactive protein, a marker of systemic inflammation is found consistently higher in all stable COPD patients. And, the level of CRP in Gold I-IV is in an increasing trend from 4.21±0.41 to 4.97±0.73 mg/L. A study showed an increased level of systemic inflammatory markers among COPD patients with MetS in the form of high-sensitivity CRP and interleukin-6.^8^ Another study noticed higher CRP levels in COPD patients with MetS. This observation indicates that the presence of MetS in COPD patients is associated with more intensive systemic inflammation.^13^ Similarly, other studies revealed higher levels of serum TNF-alpha and hs-CRP.^15^ Our finding of higher values of CRP in COPD patients with MetS is in line with the findings from other studies performed around the world. This clearly states that ongoing systemic inflammation is a phenomenon that occurs in COPD patients and the basis of this might be explained collectively by mechanisms like local inflammation spilling to the systemic compartments, smoking, hypoxia and obesity.^16^

In this study, the commonest MetS component was hypertension (90.9%), followed by altered fasting blood glucose (86.4%). In a study conducted on 1,003 COPD patients, the frequency of hypertension was 55%.^17^ Similarly, the INDACO project, a pilot study on the incidence of co-morbidities in COPD patients found 53% prevalence of hypertension in COPD patients.^18^ The results of our study showed a significantly higher prevalence of hypertension among the participants. Despite being the most common co-morbidity in COPD patients this extraordinarily higher frequency might be due to the small sample size and use of IDF criteria which classified hypertension as systolic BP >130 mm of Hg and diastolic BP >85 mm of Hg.

There were a few limitations of our study. Increasing the sample size would have made the results more reliable. And, there was an uneven distribution of the sample size in different stages of COPD. The comparison would have been statistically more significant if there was an even distribution of sample size.

## CONCLUSIONS

The prevalence of MetS in stable COPD patients was similar to the other studies done in similar settings. Treating physicians should screen all COPD patients for metabolic syndrome and intervene timely to prevent and decrease morbidities and mortalities.
